# A primer on politicization, polarization, radicalization, and activation and their implications for democracy in times of rapid technological change

**DOI:** 10.1111/bjso.12903

**Published:** 2025-05-13

**Authors:** Laura G. E. Smith, Emma F. Thomas

**Affiliations:** ^1^ University of Bath Bath UK; ^2^ Flinders University Adelaide South Australia Australia

**Keywords:** collective action, mobilization, polarization, politicization, protest, radicalization, social influence

## Abstract

Governments around the world fear a loss of social cohesion and a risk of harm to individuals and democratic processes that stem from *politicization*, *polarization* and *radicalization*. We argue that these processes of social influence provide the motivation for—but are not sufficient for—*mobilization* (the behaviour of engaging in collective action). To be able to *collectively act*, people require the capability and resources to do so, which can be developed during an *activation* process. We clarify the common and distinct aspects of each process so the common drivers, but unique effects, can be conceptualized and operationalized by policymakers, practitioners and researchers who wish to understand democratic resilience.

## INTRODUCTION

Democratic principles and institutions are under stress, leading to a fear of the loss of social cohesion and resilience (Khan, 2024). In many societies, we are more unequal (Hung, 2021) and more politically divided than we have been in the past (Winkler, 2019). Political polarization has been linked to attacks on the independence of important democratic institutions (e.g., the judiciary; Siregar, 2024) and the breakdown of civility and tolerance (Iyengar et al., 2019; but see also Broockman et al., 2023). This attitudinal polarization and hostility can lead to offline confrontation as people protest and counter‐protest (Smith et al., 2023; Smith, Thomas, et al., 2024), of course, these developments are taking place against growing global instability and a backdrop of rapid technological change. At the same time, in many jurisdictions, authorities have implemented laws that restrict some democratic freedoms (Travaglino & d'Aniello, 2023). For instance, the UK Public Order Act (His Majesty's Government, 2023a) criminalized forms of protest such as locking‐on (the protest tactic of individuals attaching themselves to others, buildings or objects), going equipped to lock‐on, obstructing major transport works, and interfering with national infrastructure.

In this *Perspective*, we explore the interconnections between social and political grievances, polarization, radicalization, and mobilization to collective action in a changing world order, as well as their interplay with policy and practitioner responses to these phenomena and digital communication technologies. Our aims are thus three‐fold: (1) to provide a conceptual overview of processes of politicization, polarization, radicalization, and activation, and their outcomes, which will (2) enable greater circumspection about potential opportunities and threats to democratic resilience implicated by the processes and outcomes, respectively, and (3) provide clarity for research, policy, and practice such that responses to these challenges can be more circumspect.

## DISENTANGLING AND UNIFYING PROCESSES AND OUTCOMES

*Collective actions* challenge a status quo (or maintain it) and are conducted on behalf of a group with which the actors identify (Becker, 2012). Tajfel (1981) formally defined collective actions as “efforts by large numbers of people, who define themselves and are defined by others as a group, to solve collectively a problem they feel they have in common, and which is perceived to arise from their relations with other groups” (p. 244). These definitions are broad in including traditional protest actions (conventional collective action), but also violent extremist actions such as terror offences (see Thomas et al., 2022). Although all intergroup action is, in one sense ‘collective action’, we limit our focus here to group actions that are designed to contest or uphold a socio‐political state of affairs (consistent with other literature on this topic; e.g. Agostini & van Zomeren, 2021).

Below, we describe these behaviours as mobilized by a set of related but distinct processes that all involve processes of social influence: politicization, polarization, radicalization, and activation. The first three are motivational psychological processes, and the latter—activation—is a more proximal process of becoming prepared and equipped for engaging in action. We conceptualize these as relatively linear, incremental (gradual) processes—to a point. Both the development of motivation and activation are likely to have a threshold, upon reaching which, people have sufficient motivation or capability/preparedness to collectively act (Bou Zeineddine & Leach, 2021; Livingstone, 2014). These thresholds are tipping points that precipitate transitioning from one behavioural state (no collective action) to another state (collective action; Livingstone, 2014).

*Politicization* involves the development of an intergroup perspective about a social or political issue alongside the perception that one's social or political grievance is shared with others, the attribution of blame to others (authorities, outgroups) and becoming involved in a contest for the support of third parties (bystanders) (Simon & Klandermans, 2001). Outcomes of this process are politicized (e.g. adversarial; intergroup) rhetoric and attitudes which form the backbone of conventional (legal, peaceful) forms of collective action (Agostini & van Zomeren, 2021; Simon & Grabow, 2010). These conventional actions are a primary way that people can hold authorities to account and raise awareness of injustice—behaviours critical for democracy to flourish (see Dalton, 2022; Thomas & Louis, 2013).

Political polarization is the process through which people extremize in their views through communication with likeminded others about their grievances (either actively through interaction on or offline, or vicariously through reading others' communications). When people extremize in opposing directions, this creates societal division. This occurs through processes of *group polarization* (Moscovici & Zavalloni, 1969; Myers & Bishop, 1970), which involves interaction about ‘how the world should be’ within opinion‐based groups (Bliuc et al., 2007) and between (oppositional) groups (Smith et al., 2015; Smith, Thomas, et al., 2024). Simon et al. (2019) argued that politicization can be an antecedent to polarization because it causes people to think more strongly in intergroup terms, although group polarization (as conceived of by Smith, Thomas, et al., 2024) is likely to also exacerbate politicization, thus making the relationship between politicization and polarization recursive.

It is easy to see how both politicization and polarization are facilitated by digital communication technologies. People use these technologies to interact and engage with each other, share and discuss grievances and intergroup perspectives, and recruit people to their cause, without the limits of physical proximity (McGarty et al., 2014). However, such interactions may increasingly involve artificially generated content that has unclear origins and, in turn, agendas: generative AI can be used to create engaging content and the messages can then be deployed through bots and bot nets, magnifying their influence (Smith, Owen, et al., 2024). To the extent that these processes occur in parallel amongst groups who support *and* groups who oppose a given position, then it creates the conditions for political or affective polarization (Smith, Thomas, et al., 2024)—intolerance and hostility between civil and political groups. Societies with a high level of such division may struggle to achieve consensus (Navajas et al., 2019).

Group‐based *radicalization* involves similar social influence processes in the sense that people develop shared social identification and commitment to collective action through social interactions with others (Smith, Blackwood, et al., 2020). Unlike politicization or polarization, however, radicalization is connected to “a change in beliefs, feelings and behaviors in directions that increasingly justify intergroup violence and demand sacrifice in defense of the ingroup,” (McCauley & Moskalenko, 2017, p. 416). Thus, radicalization can be understood as a form of polarization that involves internalizing specific norms for radical or violent collective action (Smith, Blackwood, et al., 2020; Smith, Wakeford, et al., 2020). This can be seen when members of extremist groups and opinion leaders use digital communication technologies to incite support for, and involvement in, political violence (e.g. Wakeford & Smith, 2020).

Whether or not the interactions foster violent or non‐violent political engagement depends on perceptions of the broader intergroup context in which those interactions occur (Becker & Tausch, 2015). Where groups perceive that conventional strategies cannot succeed (Saab et al., 2016; Tausch et al., 2011), that authorities will not accede to their demands (i.e. that the movement is failing; Lizzio‐Wilson et al., 2021; Louis et al., 2022), and have interacted about those beliefs with others (Thomas et al., 2014), radicalism is more likely to be seen as a necessary and justified option (Drury & Reicher, 2000). These observations suggest that the increasingly restrictive definitions of legal protest (Gulliver et al., 2023; Travaglino & d'Aniello, 2023) could paradoxically create cycles of more radical protest tactics and lower social cohesion.

It is clear, then, that authorities and those in power are therefore not bystanders of these processes, but rather play a key role in setting the terms. For example, in their counter‐extremism policies, governments decide on what is defined as extremism and therefore which groups and ideologies are included in the definition (and therefore excluded from what are considered normative or mainstream protest groups). In some cases, this has led to an increase in discrimination against members of minority groups (Blackwood et al., 2013, 2016) with implications for democratic freedoms and human rights (Human Rights Law Centre, 2024). With changes to legislation that make specific forms of protest illegal, authorities dictate what forms of collective action are acceptable (normative) and unacceptable (counter‐normative and therefore ‘radical’). This in turn, could change public perceptions of specific groups that engage in those newly illegal forms of protest (Shuman et al., 2024).

A final but critical point is that it is necessary to distinguish between motivational processes and behavioural ones. Radicalization and polarization are attitudinal and affective processes that provide the motivation for collective action, but do not necessitate engagement in action (see Khalil, 2017; Khalil et al., 2019, 2020). The attitude‐behaviour link is affected by multiple factors (see Armitage & Conner, 2001, for a review), and radical opinions are not equal to radical actions (Khalil, 2014; McCauley & Moskalenko, 2017). If they were, political violence would be more widespread (Sageman, 2004). Equally, polarization can provide the motivation for engaging in collective action (people become sympathizers; Oegema & Klandermans, 1994), but does not provide the resources and capacity to engage in concerted or effortful actions. There are a range of incentives and structural and enabling factors that affect the relationship between sympathy for particular forms of action and engagement in that action (Khalil et al., 2019). People engage with violent extremist groups for reasons other than socio‐political motivations, including financial dependence, family ties, and coerced or forced recruitment (Richards, 2014). Thus, whilst the processes of polarization and radicalization can explain an extremization of attitudes, and the expression of hateful and violent attitudes can be harmful in and of themselves, they need to be carefully differentiated (in research, policy, and practice) from the proximal predictors of engaging in action.

Politicization and polarization are thus foundational motivational processes that are necessary antecedents of collective action. Yet, to undertake collective action, people also need to become equipped with sufficient capability, know‐how, and resources to act (Brown et al., 2024; Ferree & Miller, 1985; McCarthy & Zald, 1977). We define this latter process as *activation*. Digital technologies—which enable and harness varied methods of information sharing, crowdsourcing of capability and connection—play a key enabling role (Brown et al., 2024). Once people have reached a threshold of sufficient motivation and sufficient practical preparedness, they pass a tipping point (Livingstone, 2014) and undergo a behavioural change in state (or ‘phase transition’) from not engaging in collective action to engaging in collective action.

Whilst engaging in collective action requires individuals to go through both a motivational process and one of activation, engaging in other forms of coordinated, group‐based action (for example, responding to emergencies or disasters, see Perach et al., 2023) may require people to pass only an activation tipping point. One can engage effectively in coordinated, group action if one has the capability/resources, but without internalizing a socio‐political motivation for that action. At the same time, people participating in coordinated group action in response to an event such as an emergency could become politicized and polarized, and thus internalize socio‐political motivations for collective action.

Collective action (as we have defined it here) is about group actions to change socio‐political systems. Other forms of group‐based action (e.g. volunteerism, or benevolent support for groups experiencing disaster or emergency; Perach et al., 2023; Thomas & McGarty, 2018), which are not necessarily accompanied by a politicized and/or intergroup awareness, could become activated directly. At the same time, some group members may have a politicized understanding of the disaster or emergency—for example, that aid is unequally distributed. For these people, attributions about an unequal system of intergroup relations (politicization) and an us‐them consciousness (polarization) mean that they have internalized more socio‐political motivations (see Thomas, Bird, et al., 2024; Thomas, Yip, et al., 2024). It is also the case that people may engage with a benevolent (non‐politicized) standpoint, but the process of taking action may itself be a politicizing and polarizing experience (Drury & Reicher, 2000; Vestergren et al., 2017).

Given that some people engage in group action for reasons other than socio‐political motivations (Amiot et al., 2020; Thomas, Yip, et al., 2024; Yip et al., 2025), there is likely to be a bidirectional relationship between engaging in action and the development of motivation. For example, engaging with the practicalities of gathering resources and know‐how for collective action could facilitate the development of the motivational processes. We must therefore draw distinctions between collective action that is socio‐politically motivated and other forms of coordinated group‐based action that are not (see also Thomas, Bird, et al., 2024; Thomas & McGarty, 2018; Thomas, Yip, et al., 2024). Furthermore, we assume that people can engage in socio‐political collective action without being sufficiently prepared (activated). The effectiveness and motivational basis of action that occurs as a result of this set of processes will therefore depend upon the extent to which individuals undertaking that action reach the thresholds for socio‐political motivation and preparedness.

Our arguments are consistent with a *complex systems approach* that sees participation in collective action as part of a multi‐level, dynamic, recursive system (Bou Zeineddine & Leach, 2021; Thomas et al., 2022). Whilst here we focus on tipping points for the behaviour of individual group members, collective action emerges in a complex sociotechnical system, involving the spread of ideas via digital technologies, and the actions of state actors, non‐governmental organizations, authorities and individuals. Changes in any of these aspects could tip the system in a way that motivates (members of) a group to take collective action. By taking a complex systems view, we can start conceptualizing and predicting how, when, and why people and societies transform through these processes.

Critically, these points imply the need to adopt different methodologies into our toolkit to study non‐linear changes. For example, person‐centred methods like latent profile analysis can capture the discrete sub‐groups of people who are qualitatively different in their reasons for engaging in action, as well as model their transition from one subgroup to another (Thomas, Yip, et al., 2024). To gain insight into the factors that ‘tip’ a system from one state to another (Nowak & Vallacher, 2019), agent‐based models (ABM; e.g., Fennell et al., 2023) are perhaps uniquely well‐suited to simulating the emergent dynamics that arise from interactions within and between people, groups (movements), authorities and antagonistic outgroups. For example, ABMs have been used to show that distinct opinion‐based groups can emerge from an initially homogeneous population via social interactions (Flache et al., 2017; Noorazar, 2020). The end state of the system depends on factors such as structural concerns (Smaldino et al., 2012), the frequency of interactions between ingroup and outgroup members (Carpentras et al., 2023), and technological factors like algorithms and recommender systems (Geschke et al., 2019). Thomas et al. (2025) report an empirically informed agent‐based model which reveals that severe state repression of protest (i.e. high levels of protest failure, inability to contest the failure) leads to a population who are inactive but radicalized—and prepared to use radical tactics once environmental conditions change. A change in the prevailing conditions would tip the system into another state.

These methods provide promising avenues by which psychologists can study and predict non‐linear collective behavioural phenomena like the emergence of new social movements, the start of mass protests or the outbreak of conflict that are precipitated by linear changes in motivational processes (Livingstone, 2014; Smith et al., 2019). This opens up new possibilities for theorizing and modelling the start and end points of societal changes, and the conditions and thresholds that are necessary and sufficient for tipping people, groups, and societies from one state to another.

## IMPLICATIONS FOR RESEARCH, POLICY AND PRACTICE

Our analysis highlights the tensions between, on the one hand, repression of collective action as an ostensible driver of division (polarization) within democracies and, on the other, the role that such repression may play in bringing about the suppression of human rights, erosion of liberal values and radicalization. Clarity on the definitions of these concepts and how to operationalize them should help ensure that investigations or attempted mitigations of threats to democracies or promotion of civic engagement engage with these tensions in mind. Indeed, awareness of differences between the phenomena should help practitioners and scholars to pinpoint precipitating conditions and moderators for each outcome.

These complexities run deeper still because political engagement and (conventional, non‐violent) protest are critical for democracy to flourish (Dalton, 2022). Yet, there is also evidence that this can be weaponized to disrupt social cohesion and incite civil conflict as part of a broader campaign to destabilize democracies in a changing world order (Cosentino, 2020; Vićić & Gartzke, 2024). Cosentino (2020) provides examples of protests and counter‐protests that were created and organized by the Russian Internet Research Agency as part of a strategy to interfere in political processes and outcomes (so‐called information warfare). Therefore, authorities (i.e. governments, legislators, politicians, police) need to strike a balance between enabling processes that promote conventional forms of political engagement and managing the social disruption that (occasionally) emerges from such engagement, including from the use of technologies by malicious actors to influence social cohesion. Much of the literature addressing these processes does so through the lens of mis/disinformation, information operations, and/or conspiracy beliefs and is, as yet, largely disconnected from the literature/s on politicization, polarization, radicalization and activation—although some work connecting conspiracy beliefs and extremism has begun to emerge (see e.g. Imhoff et al., 2021; Jolley et al., 2022; Pummerer, 2022; Rottweiler et al., 2022; Thomas, Bird, et al., 2024). A more complete response will embed the effects of mis/disinformation and/or conspiracy beliefs in the dynamics of influence and collective action identified here. Accordingly, based on these insights, we make four suggestions for practitioners and researchers.

First, it is critical to appreciate that the processes described above continue and evolve over time – including changes in the forms of action that groups decide to take (Lizzio‐Wilson et al., 2021; Louis et al., 2022). An event (e.g. a protest) is not an endpoint but rather an input to the ongoing socio‐structural and political dynamic. Although the research effort is nascent, criminalizing (what were hitherto legal) forms of collective action seems unlikely to deter people with grievance (see Gulliver et al., 2023), and may create fertile ground for the emergence of more disruptive tactics (Louis et al., 2022). Research on the policing of crowds and protest dynamics is especially instructive here: Drury, Reicher, Stott and colleagues have shown that perceived indiscriminate and disproportionate police reactions to what was primarily conventional protest can radicalize protesters (e.g. Drury & Reicher, 2000), and lead to spread (e.g., Drury et al., 2022). Indeed, as we have suggested above, political violence is more likely to emerge amongst group members who do not feel that conventional alternatives are a viable option. Therefore, whilst legislating against violent protest and the incitement of hatred and violence online is beneficial (e.g. in providing authorities with the power to intervene when members of extremist groups seek to harm others), criminalizing certain forms of (previously legal) protest may increase risk in some cases (Thomas et al., 2025). Rather than being a deterrent, it may lead to groups considering more harmful tactics than they have done so before.

Second, whilst risk assessment of individuals in counter‐terrorism approaches may audit the social and group factors that impact an individual's risk, they do not address the social influence processes described here (i.e., those processes that occur within groups and during interactions, rather than within individuals). Indeed, there are concerns about the risk assessment tools used by the Channel and Prevent multi‐agency panel programme in the United Kingdom (Home Office, 2023), and community safeguarding in Australia (Australian Government, 2022) (Corner & Taylor, 2023; Smith, Blackwood et al., 2020; Smith, Owen, et al., 2024). Whilst extremist groups can be proscribed, such approaches rarely address the group processes of social influence that create the motivation for violence. And whilst some risks created by social influence processes that unfold via digital communication technologies are implicitly recognized in legislation such as the UK Online Safety Act (which places a duty on digital communication technology companies to risk assess, mitigate the impacts of, and remove illegal content; His Majesty's Government, 2023b), the 2024 riots in the UK demonstrated the need to better manage legal but harmful communications that can motivate people to radicalize—for example, misinformation that uses politicizing intergroup rhetoric and undermines trust in authorities (The Guardian, 2024).

Revised risk assessment processes implemented as part of a counter‐extremism strategy could seek to better understand the group context within which individuals interact, addressing the (online and offline) network that enables their activation. Within groups of interest, some people may play a radicalizing role, focusing on fostering ingroup consensus on grievances and the incitement of violence, and some other people may play an activating role, focusing on resources, and organizing and planning a group event. Others may not take either role but may become collective actors because of the influence of others. By auditing the heterogeneity of roles within a group, risk assessments can account for both individual and group‐level processes and may be more equipped to accurately assess and manage risk. Indeed, focusing only on individuals without appropriately assessing relevant group processes misses potential transformative intelligence. Taking a complex systems approach to understanding group radicalization raises novel empirical questions such as how to reverse a state or behavioural change. Deradicalization may not occur simply by removing risk factors of radicalization for a group; the deradicalization process would occur under a different set of conditions to the radicalization process, and therefore may need to pass different tipping points to return to what is considered a ‘baseline’.

Third and relatedly, by understanding terrorist action as a collective action that requires activation, it becomes apparent that risk assessment procedures must not (only) look for an intensification of expressed motivations for such actions, but also the activation process (Brown et al., 2024), including signs of the resource‐based, non‐volitional, and instrumental reasons people may become involved in action. This should be approached as a group‐based risk assessment procedure (i.e. the risk indicators may not be apparent if one assesses an individual, but they may be apparent at a group level).

Fourth, and more broadly, to understand (changes in) social cohesion, these processes can be modelled using communications data from social interactions and the metadata surrounding them. For accuracy, the operationalization of the concepts should be precisely aligned with their definitions and validated through research. For example, social media data has been used to model the expression of grievances (van der Vegt et al., 2021), extremization of individuals (for polarization and radicalization) (Smith et al., 2023; Smith, Thomas, et al., 2024), group polarization (Del Vicario et al., 2016), political polarization (Falkenberg et al., 2022), activation (Brown et al., 2024) and mobilization (Smith et al., 2023), respectively. Practitioners can take advantage of the opportunities proffered by advances in natural language processing, machine learning and AI‐based collective intelligence systems to model these processes as they unfold, and to support decision making.

Finally, we should consider the relationship between empirical research and authority structures, and the roles that social psychologists can play. There are those who would seek to use social psychological insights to damage social cohesion and democratic resilience. We can advise authorities on how to protect against this hostile interference, and enhance social cohesion and democratic resilience, but we can also highlight how politicizing particular groups and psychological phenomena in policies and legislation can produce and perpetuate grievance—paradoxically spurring the dynamics that authorities seek to avoid. Researchers of these processes should be aware of potential dual‐use concerns (Smith, Owen, et al., 2024), and engage with frameworks that help to anticipate and avoid unintended and potentially harmful applications of psychological research (Stilgoe et al., 2013).

## CONCLUSION

Democracy faces a perfect storm of increasing grievance, social and political change, technological advancement and growing global instability. In the face of this storm, to protect democratic freedoms and social cohesion, policy, practice, and research need to carefully disentangle and understand the processes and predictors of the phenomena involved in mobilization: politicization, polarization, radicalization and activation, as well as the risks, threats, and opportunities that digital technologies pose. The aim of doing so is to ensure investigations and mechanisms that aim to safeguard democracy can be most appropriate, whilst recognizing that democratic resilience and maintaining peace involve protecting the right to, and behaviour of, protesting and free speech.

## AUTHOR CONTRIBUTIONS

**Laura G. E. Smith:** Conceptualization; writing – original draft; writing – review and editing. **Emma F. Thomas:** Conceptualization; writing – original draft; writing – review and editing.

## CONFLICT OF INTEREST STATEMENT

Both authors have no conflicts of interest.

## Data Availability

There are no data associated with this Perspective article.
